# Golden oldies: ten crystallography articles that we think must be read^1^

**DOI:** 10.1107/S2056989023004619

**Published:** 2023-06-06

**Authors:** Chiara Massera, John R. Helliwell

**Affiliations:** a Dipartimento di Scienze Chimiche, della Vita e della Sostenibilità Ambientale, Università di Parma - Viale delle Scienze 17/A, 43124 Parma, Italy; b Department of Chemistry, University of Manchester, M13 9PL, United Kingdom; Universidad de Los Andes Mérida, Venezuela

**Keywords:** landmark papers in crystallography, science pull, technology push

## Abstract

We have selected a set of ten ‘golden oldies’, diverse crystallography articles to illustrate important moments in the development of our field of science and which form landmark papers in crystallography. They are a mixture of ‘science pull and technology push’. For each of our choices, we firstly created a new title that emphasizes how the paper’s importance worked out from today’s perspective. Then we describe the core details and impacts of each paper, with some quotations and a selected figure or two. Ten is an arbitrary number of highlights and our choice is personal.

## Abstract 2

We have selected a set of ten diverse crystallography articles to illustrate important moments in the development of our field of science. They are a mixture of ‘science pull and technology push’. Ten is an arbitrary number and our choice is personal, so many others might have been chosen.

## Introduction

We should explain first why we wrote this article? One of us (CM) had the idea and invited JRH to join in, within the recent initiative of the *Acta Crystallographica Section E* Main Editors to publish a series of educational articles, of which this would be one. We had already discovered our similar approaches to our science, of using multiple methods to move beyond the precision of one method alone (crystallography) and reach accuracy, when we both taught on the European Crystallography School 6 (‘ECS6 Budapest’). Yet our areas of science were distinctly different, smaller and bigger molecules, respectively, and thereby nicely complementary for this article. We had also worked well together recently on another education article in *J. Appl. Cryst.* (Helliwell & Massera 2022 ▸).

The choice of articles is inevitably a personal one. We firstly opted for those authored by individuals who are no longer with us. This is a neat mechanism to minimize the risk of losing our friends. Secondly, a maximum of ten articles is going to be woefully inadequate, so we foresee a series of future articles from colleagues who will make their own top ten selections. As our overall aim is education, with a historical focus, we draw attention to the article by Jenny Glusker (1998 ▸) ‘*The teaching of crystallography: A historical survey*’; her selection of refer­ences provides an independent suite of examples.

For each of our choices we firstly created a new title that emphasizes how the paper’s importance worked out from the perspective of today. Then we describe the core details and impacts of each paper, with some quotations and a selected figure or two.

If we address the question: ‘what causes progress, science pull or technology push?’ Undoubtedly both. The discovery of X-rays by Roentgen, and then the demonstration of X-ray diffraction by a crystal by Friedrich, Knipping & von Laue (1912 ▸) created a technology push to the Braggs to solve the first X-ray crystal structures. Later, Banerjee’s theoretical formalism for phasing, namely the determination of the signs of a centrosymmetric crystal’s structure factors, was science pull but way ahead of its time since there was no computer available to exploit it. Likewise, the Okaya & Pepinsky (1956 ▸) formalism to vary the X-ray wavelength to solve the phase problem was impeded by the lack of a synchrotron radiation source, but its thinking truly was a gem. We also refer you to Peerdeman (1975 ▸) for a broad historical perspective of the crystallography community thinking from the 1940s onwards on the importance of anomalous dispersion in crystal-structure analysis, starting with Bijvoet (1949 ▸), including ‘the use of more than one wavelength’. Bernal & Crowfoot (1934 ▸) worked out that trying to measure X-ray diffraction from a protein crystal by a usual dry mount was not the way to do it, and instead showed that protein crystals must be kept moist [allegedly following an original idea of Megaw (1934 ▸)], and so the whole new field of biological crystallography commenced. For structure and bonding studies to flourish, *i.e.* the field of structural chemistry, model refinement against the measured diffraction data was needed. This was technology push as new electronic calculating machines made researchers think how they could be used; Hughes (1940 ▸, 1941 ▸) documents that transition from using Beevers–Lipson strips to an IBM calculating machine. The field of charge-density analysis could also develop and thrive owing to constant improvement of the data quality driven by more powerful X-ray sources, detectors, and low-temperature equipment. We are very conscious that in our article we are leaving out Patterson (1934 ▸), whose work opened up the determination of interatomic vectors between atoms, from which, when combined with possible symmetries of crystals and thereby knowing the Harker sections, atomic coordinates could be determined. However, with him being such a famous crystallographer, and the Patterson synthesis also, Patterson (1934 ▸) will be read in any case. Anyway, in imagining the development of crystallography as a sort of phylogenetic tree there are so many branch points. There were also practices that were advanced communally or collectively. First and foremost, as we highlight in our companion education article (Helliwell & Massera, 2022 ▸), trust in crystal structures stems from constant application of the *checkCIF* utility and the PDB validation report, developed by extensively discussing topics and challenges together in IUCr Journals and the IUCr Commissions, as well as the PDB’s Expert Advisory Groups.

Since X-ray production technology appeared first, the use in crystallography of our two other diffraction probes, electrons and neutrons, appeared later. Maybe inevitably they were to inherit a role of working around the edges, *i.e.* perhaps viewed as filling in the gaps left by X-ray crystal-structure analysis alone. For electrons, ultra-small or very thin crystals became their domain for structure determination, as described in detail in the book by Boris Vainshtein (1964 ▸). His descriptions (pages 300–307 of his book) of crystal structures determined by electron diffraction commenced with a detailed description of structural studies of BaCl_2_·H_2_O. Moving on to neutrons, their domain for crystal-structure analysis became focused on magnetic structures or the structures where hydrogen atoms were of interest, including hydrates. In the book by George Bacon (3rd edition 1975 ▸), his descriptions of molecular structures determined by neutron diffraction commenced with a detailed description of the work by Garrett (1954 ▸) on oxalic acid dihydrate. The contoured nuclear density map, that revealed the oxygen atom of a water molecule as a positively contoured peak and its two hydrogens as negatively contoured peaks, is a further elaboration of W. L. Bragg’s description of a crystal structure as being able to see atoms (Bragg, 1968 ▸). The positive and negative contours occur due to the scattering signatures for neutrons of oxygen and hydrogen, respectively.

Within the caveat of the above opening remarks, we move on now to describe our selected top ten articles and their roles within our perspectives. We follow a historical timeline of:

Ewald, 1969 ▸ (but which is really ∼1912 to 1917)
W. L. Bragg, 1913 ▸
Lonsdale, 1929 ▸
Banerjee, 1933 ▸
Megaw, 1934 ▸
Bernal & Crowfoot, 1934 ▸
Bijvoet *et al.*, 1951 ▸
Okaya & Pepinsky, 1956 ▸
Coppens, 1977. ▸

## P. P. Ewald (1969 ▸). *Reciprocal space and Ewald’s sphere*

**Figure 1:**
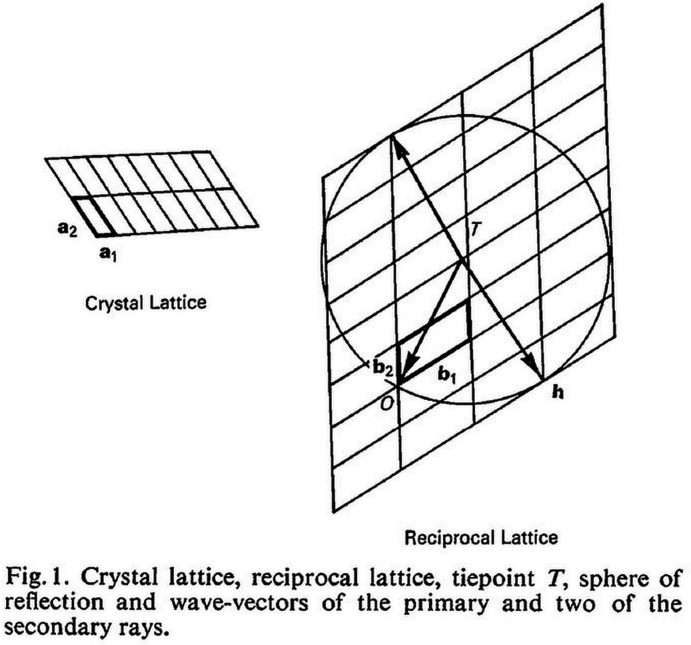
Description of the geometrical theory of diffraction. Reproduced with permission of the International Union of Crystallography.

From the beginning of his scientific career, Paul Ewald had been interested in the propagation of electromagnetic radiation in crystals. Already in his PhD thesis, concerning the propagation of visible light, he postulated that the dipoles excited by the light in the crystals generate an electromagnetic field: self-consistent, independent from the incident radiation and determined by the crystal structure. His studies, which eventually led to the dynamical theory of X-ray interferences [declared by von Laue as ‘a masterpiece of mathematical physics’ (Bethe & Hildebrandt, 1988 ▸)], gave support to the idea that crystals were an ordered and periodic distribution of atoms or molecules and brought with them the now universally known concepts of the reciprocal lattice and the ‘sphere of reflection’ (the so-called Ewald’s sphere). Although in X-ray diffraction experiments most of the time mosaic crystals (Darwin, 1941*a* ▸,*b* ▸ ) follow the kinematical theory of scattering (in which multiple scattering effects in the interactions of X-rays with matter are neglected), the applications of the dynamical theory are numerous. It is used in electron diffraction, in ‘perfect’ crystals often related to the semiconductor industry, and in the design of monochromators, to cite just a few (Authier, 2006 ▸). Against this background, Ewald’s 1969 ▸ paper ‘*Introduction to the Dynamical Theory of X-Ray Diffraction*’ is particularly didactic; besides dealing with the description of the dynamic effects and their connection to the general theory of small oscillations of a mechanical system, it gives a very clear summary and overview of the different theories of diffraction, and of the concept of Ewald’s sphere. This concept, which is used to describe graphically the so-called ‘geometrical theory’ of diffraction and to establish the direction of the diffracted beams, is represented in Fig. 1 ▸. The sphere of reflection of radius 1/λ (λ = wavelength of the radiation) has its centre at *T*, the ‘tie-point’ that ties together the vector *T*O of the incident wave and the vector *T* **h** of the diffracted wave, which forms whenever the sphere passes through a point **h** of the reciprocal space. Moving to the kinematical theory, ‘*the combined effect of the scattered wavelets in directions other than those of maximum cooperation is taken account of*’. Since we are dealing with finite crystals limited by an external boundary, their scattering power is represented by a Fourier integral, and not by a Fourier series as would be the case in a truly periodic crystal. In Ewald’s words, ‘*the purely mathematical and physically unreasonable ‘all or nothing’ condition of intersection of sphere of reflection and lattice point is now replaced by the more generous result of the superposition of the elementary wavelets*’.

The rest of the paper focuses on the dynamical theory of diffraction which, unlike the kinematical theory, takes into account all the multiple scattering of X-rays. Ewald had already developed this theory back in *ca* 1912–1917 but at the time, and for the following years, it had not found many applications due to the ‘faulty’ nature of real crystals, which are never single but always show a certain degree of mosaicity. The theory ‘*…came into prominence when the art of growing perfect or near-perfect crystals was developed, and when the discussion of electron diffraction […] demanded some such theory because of the much stronger interaction of matter with electrons than with X-rays*’.

*Brief biographical sketch:* Paul Peter Ewald (1888–1985) was a German scientist best known for his dynamical theory of crystallography and for the so-called Ewald sphere, a geometric construction that explains the occurrence of diffraction spots and determines the angles at which the Bragg equation is satisfied. Ewald received his doctoral degree in 1912 from the Ludwig Maximilian University of Munich, where he remained for a period as Privatdozent, and assistant to Arnold Sommerfeld, who had formerly been his supervisor. He later became a Professor at the Technische Hochschule Stuttgart, but due to problems with German National Socialism, he emigrated to the UK, and accepted a position at Queen’s University Belfast in 1939. Ten years later he moved to the Polytechnic Institute of Brooklyn, where he remained until the end of his career. It is worth remembering that in 1944 he proposed the establishment of the International Union of Crystallography, of which he became the president in 1960. In his honour, the IUCr created the Ewald Prize, to be awarded for outstanding contributions to the science of crystallography (https://www.iucr.org/iucr/ewald-prize). A memoir of Prof­essor Ewald can be found at https://royalsocietypublishing.org/doi/pdf/10.1098/rsbm.1988.0006.

## W. L. Bragg (1913 ▸). *The first X-ray crystal structures*

**Figure 2:**
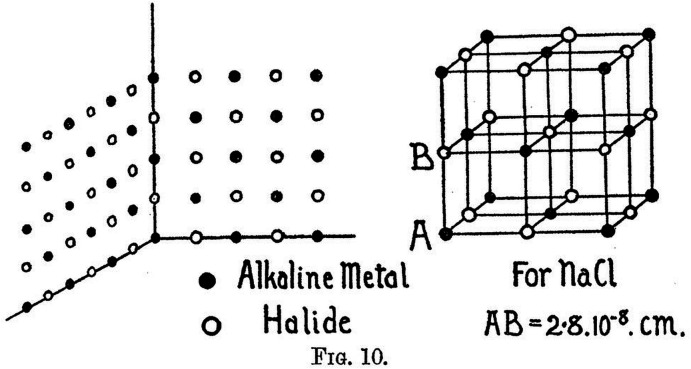
The three-dimensional structure of the alkali halides (Bragg, 1913 ▸). We acknowledge the *Proceedings of the Royal Society*.

Perhaps the most incredible paper of the ten we have chosen is this one. By comparing the Laue diffraction patterns of several alkali halide crystals (KCl, KBr and NaCl) measured by Lawrence Bragg at the Cavendish Physics Laboratory in Cambridge, he deduced their crystal structure. From the modern day we can see that it is in effect an isomorphous replacement between the three crystals. In addition, it presents the underpinning raw diffraction data in the 15 Laue diffraction photographs. The patterns of diffraction spots in each of these depict the intensities schematically as well as providing their Miller indices. The fulcrum of the analyses is that for the KCl crystal, the atoms of potassium and those of chlorine have practically equal scattering powers and so a primitive cubic lattice is the interpretation. For NaCl and KBr, the spacing of the lattice is doubled, deduced by the fact that their Laue diffraction patterns are more complex. In his career look-back book ‘*The Development of X-ray Analysis*’ (Bragg, 1975 ▸, page 30) he reminisces that:

*‘It was on this rather indirect and slender evidence that I assigned the structure of Figure 12* (Fig. 2 ▸ here) *to the alkaline halides in a paper read to The Royal Society in June 1913; fortunately, further investigation established its correctness!’*

Then on pages 33–34 of Bragg (1975 ▸):

‘*Measurements of these spectra* [with his father’s monochromatic X-ray spectrometer] *provided a far more powerful way of examining crystal structure. For instance, the solution of the sodium chloride structure, so laboriously deduced from the Laue photographs, is now seen at once from the X-ray spectrometer measurements of X-rays reflected from a sodium chloride crystal from the (100) face and from the (111) face.’*

At the time it was thought that the scattering power of an atom was proportional to atomic weight and was later modified to be proportional to atomic number. The distance between the alkali halide atoms (2.8 Å for NaCl as shown in Bragg’s figure above, Fig. 2 ▸) was too large for a chemical bond and so it was realized that the layout of the atoms was one of alternating positive and negatively charged ions. Thus K^+^ and Cl^−^ were isoelectronic as far as the X-rays were concerned.

*Brief biographical sketch*: William Lawrence Bragg (1890–1971) was an Australian-born British physicist. He was awarded the Nobel Prize in Physics in 1915 along with his father William Henry Bragg (1862–1942) ‘*For their services in the analysis of crystal structure by means of X-rays*’. There is an extensive biographical memoir of W. L. Bragg (Phillips, 1979 ▸) and of W. H. Bragg (da Andrade & Lonsdale, 1943 ▸). It is very important to note that Lawrence Bragg and his father reacted incredibly promptly to the discovery of the diffraction of X-rays by Friedrich, Knipping and von Laue in 1912 ▸ in Munich by making their own investigations and developments. It is also interesting to wonder that Roentgen in 1895 placed various items, but not a crystal, in the X-ray beam emanating from his vacuum tube. Laue, stimulated by a discussion with Paul Ewald, had the insight to imagine that a crystal might behave as a diffraction grating for X-rays. The early days of X-ray crystallography are described in detail in the book by André Authier (2015 ▸).

## K. Lonsdale (1929 ▸). *The determination of the planar structure of benzene*

**Figure 3:**
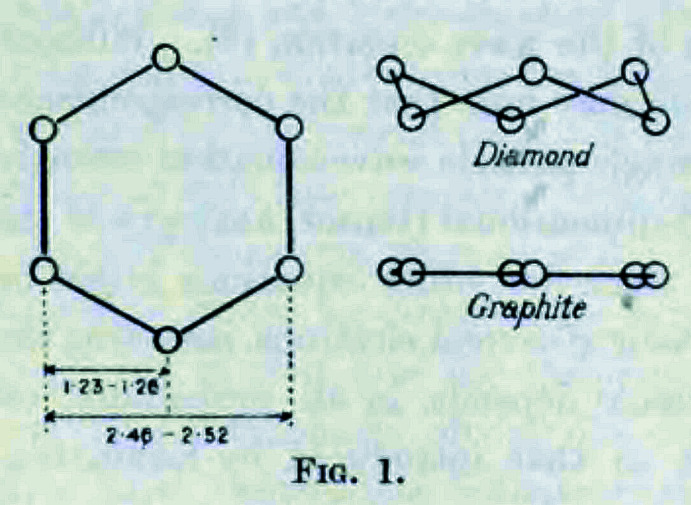
This figure is as found in the original paper, showing the two possible arrangements of carbon atoms in benzene. We acknowledge the *Proceedings of the Royal Society*.

Going through this 21-page-long paper is like reading a mystery novel, where the final revelation is not the identity of a murderer, but the spatial arrangement of atoms in benzene. The reader is immediately presented with the relevant clues available from previous investigations: the six carbon atoms of benzene are probably disposed in a centrosymmetric ring, which shows an approximate width of 2.49 Å and two periodicities of 1.28 and 2.66 Å, respectively. Two possible situations match these experimental data: either the carbon atoms form a puckered ring as in the structure of diamond, with a ring width of 2.52 Å and periodicities of 1.26 and 2.52 Å, or they are arranged in a planar hexagon as in graphite, with a ring width of 2.46 Å and periodicities of 1.23 and 2.46 Å (Fig. 3 ▸).

The paper shows step by step the deduction process which, by the analysis of the diffraction data from the crystals of hexamethylbenzene, gives the answer to this conundrum. The key role of this benzene derivative derives from two great advantages: it is solid at room temperature, and its triclinic unit cell contains one molecule only, thus eliminating the problems of relative orientations.

### Step 1: Structural investigation

The X-ray diffraction on prismatic crystals of C_6_(CH_3_)_6_ grown from benzene yielded the following unit-cell values: *a* = 9.010 Å, *b* = 8.926 Å, *c* = 5.344 Å, α = 44°27′, β = 116° 43′ and γ = 119° 34. Due to the similar lengths of *a* and *b*, and to the γ angular value being very close to 120°, Lonsdale’s first approach was to find evidence of a hexagonal arrangement of the carbon atoms by examining the diffraction pattern of the [001] zone. This was demonstrated by the similarity of the structure factors recorded for different planes, grouped in three families: those lying between (100) and (010), between (0 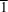 0) and (1 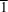 0) and between ( 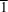 10) and ( 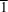 00). It was also found that the intensities from the (001) cleavage plane fell off regularly in the first four orders, similar to what happened in the corresponding cleavage plane of graphite.

### Step 2: Location of the carbon atoms

**Figure 4:**
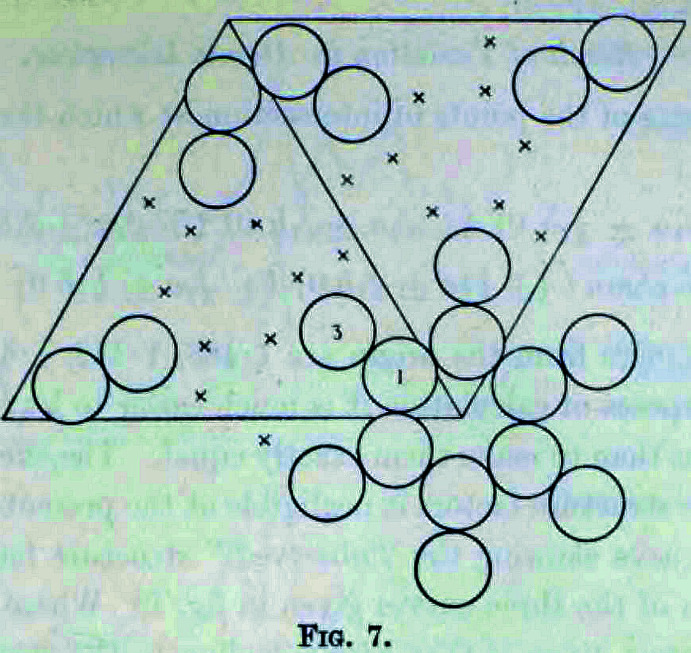
This is Figure 7 of the original paper and indicates the positions of the carbon atoms; once two atoms were located in positions 1 and 3 by analysing the structure factors, the other ten corresponding positions were obtained by rotation of 2π/6 about the *c* axis. We acknowledge the *Proceedings of the Royal Society*.

The location of the carbon atoms was found by combining the information provided by the values of the structure factors with the necessity of obeying the symmetry requirements of a hexagonal structure (Fig. 4 ▸).

This was the first time that an aromatic substance ‘…*had a simple enough structure for the positions of the separate atoms to be found without any previous hypotheses as to the shape or size of the molecule*’. The positions thus determined yielded an average distance from centre-to-centre of adjacent carbon atoms in the molecule of 1.48 Å, a mean value between the carbon-atom diameters in diamond (1.54 Å) and in graphite (1.42 Å). The next step was therefore dedicated to determining the atomic positions and diameters more exactly.

### Step 3: Exact determination of atomic positions and diameters

In this last part of the paper, the effect of three geometrical variations on the structure factors were analysed.

(i) Variation in the atomic diameters and consequently in the size of the ring. In this case the best agreement was obtained when the diameter of the aromatic carbon atoms was 1.42 Å, as in graphite.
(ii) Rotation of the ring in the (001) plane. Overall, the rotation brought a mismatch between calculated and observed structure factors.
(iii) Shifts of the atoms perpendicular to the (001) plane, *i.e.*, a puckering of the benzene ring and methyl groups. Also in this case, the agreement between calculated and observed structure factors was maintained only if both the nuclear and the side-chain carbon atoms remained within 0.1 Å from the (001) plane.

In the conclusions of the paper, Lonsdale establishes that ‘*the puckered or ‘diamond’ type of benzene ring and the more compact model suggested by J. K. Morse are shown to be wholly inadmissible*’. To the modern reader, it is a beautiful and neat example of a solution process carried out through a rigorous analysis of the structure factors.

*Brief biographical sketch*: Dame Kathleen Lonsdale, née Yardley, (1903–1971) was the Irish crystallographer who first determined the structure of an aromatic compound (hexa­methylbenzene), proving that the benzene ring was flat and determining its geometrical parameters. Born in County Kildare, she moved with her mother and siblings to the UK, where she eventually graduated in physics from University College London in 1924. Her scientific crystallographic career was mainly carried out between the University of Leeds and the Royal Institution in London, where she worked with W. H. Bragg and where she discovered the structure of benzene and its derivatives. From 1949 until her retirement, she became Professor of Chemistry and Head of the Department of Crystallography at University College London. She was the first woman to be elected President of the International Union of Crystallography (1966) and President of the British Association for the Advancement of Science (1967). Memoirs and papers dedicated to Dame Kathleen’s work and life (Hodgkin, 1975 ▸; Wilson, 2015 ▸) can be found at: https://royalsocietypublishing.org/doi/epdf/10.1098/rsbm.1975.0014, https://www.tandfonline.com/doi/full/10.1179/0308018815Z.000000000117 and https://discovery.ucl.ac.uk/id/eprint/1573430/.

## K. Banerjee (1933 ▸). *The first description of a direct method of phasing*

This article was communicated to the Royal Society by William Henry Bragg. It was entitled *‘Determination of the signs of the Fourier terms in complete crystal structure analysis’.* Recall that the start of the era of direct methods of phase determination was credited to Karle and Hauptmann (https://www.nobelprize.org/prizes/chemistry/1985/summary/) ‘*for their outstanding achievements in the development of direct methods for the determination of crystal structures*.’ Of course, the paper of Banerjee (1933 ▸) was one of those ‘ahead of their time’, not least because the computer had not been invented in 1933. The Nobel Prize Award Ceremony speech (https://www.nobelprize.org/prizes/chemistry/1985/ceremony-speech/) made no reference to Banerjee, but Jerry Karle in his Nobel Lecture article (Karle, 1985 ▸) did cite Ott (1928 ▸) and Banerjee (1933 ▸): ‘*There were some early attempts to obtain structural information or phase information from the structure factor equations. Ott (1928 ▸) made use of the structure factor equations to derive relationships among the structure factors and atomic positions and he showed that in some simple cases atomic coordinates could be obtained directly from the relationships. Banerjee (1933 ▸) devised a trial-and-error self-consistency routine based on Ott’s results for finding the phases of structure factors that are centric and therefore with phases that have values that are limited to zero or π. The number of trials increased rapidly with complexity limiting applications to rather simple structures*.’

**Figure 5:**
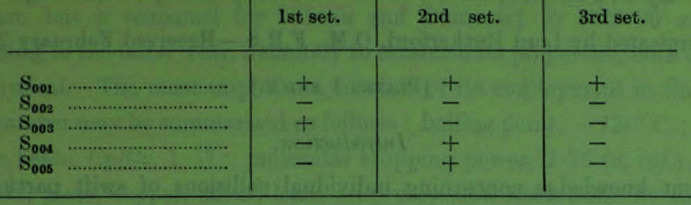
The signs of the (00*l*) reflections of anthracene in three possible phase sets (from Banerjee, 1933 ▸). We acknowledge the *Proceedings of the Royal Society*.

The paper, Banerjee (1933 ▸), starts by providing an overview of crystal-structure determination of the time, referring to W. L. Bragg’s approach as one of trial and error, which it was, and likewise used by all in the field. The trial-and-error approach was guided by any information that could also be harnessed, be it data on the chemical or physical properties. Banerjee (1933 ▸) also pointed out that W. H. Bragg (1915 ▸) had introduced the Fourier method to crystal-structure analysis. Thereby, with a rough trial-and-error first structure, the signs of the terms in the Fourier series could be calculated ‘*and the complete analysis effected by the Fourier method*’, the signs being appropriate for the case of a centrosymmetric structure. Banerjee (1933 ▸) introduces his new method with the comments ‘*All the methods previously described are applicable only to simple crystals, or to crystals whose physical and chemical properties are a great help in determining the structure. Most crystals are thus too difficult to yield to these methods, e.g., the aromatic organic crystals, except a few isolated cases.’* He develops a set of equations, in the centrosymmetric case, ‘*a series of equations whose number depends on the number of intensity measurements available. With the help of the signs that have already been determined it is now possible to find the signs of these terms from these equations.’* He introduces the terminology in an example (anthracene, and the (00*l*) reflections) of possible phase sets (Fig. 5 ▸).

Banerjee (1933 ▸) validates his solution ‘1st set’ from the estimates based on trial-and-error structure signs, but concludes his paper by stating ‘*But the real usefulness of the method will be in rather complicated cases, where the method of trial and error fails, though a good series of intensity measurements has been made.’*

Banerjee concludes his article with an acknowledgement to ‘J. M. Robertson for letting him have access to the unpublished X-ray intensity measurements for anthracene’. This was published (Robertson, 1933 ▸) and Table 1 in that paper shows the calculated values for the reflections, including the (00*l*) series and their signs are as given in Banerjee’s phase set I.

*Brief biographical sketch*: Kedareswar Banerjee (1900–1975) was an X-ray crystallographer and Director of the Indian Association for the Cultivation of Science, Kolkata. Early in his career he determined the structures of naphthalene and anthracene. In 1931, he worked with Sir William Henry Bragg and developed one of the first direct methods of crystal-structure determination. He was Professor of Physics at the Indian Association for the Cultivation of Science from 1943 to 1952 and Director of the Association from 1959 until his retirement in 1965. In 2000 there was a centennial celebration held in Kolkata in honour of the memory of Banerjee’s contributions to the development of crystallography in India. At this centennial, JRH learnt about his 1933 ▸ paper.

## H. D. Megaw (1934 ▸). *The nature of ice and the importance of collecting crystals in their mother liquor*

**Figure 6:**
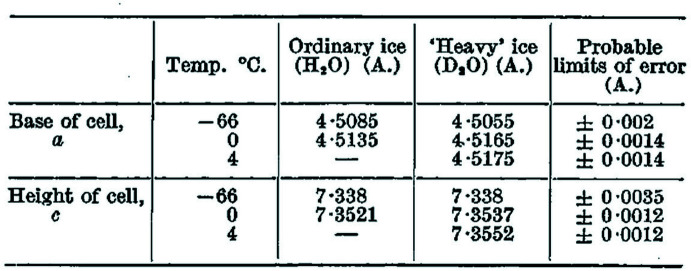
Table 2 from the Nature paper by Megaw (1934 ▸) showing the comparison between the cell dimensions of ordinary and heavy ice. From Megaw, H. D. (1934 ▸). *Cell Dimensions of Ordinary and "Heavy" Ice*. *Nature*, **134**, 900–.901. Copyright Springer Nature. Reproduced with permission.

This paper appeared as a Letter to Nature reporting the ‘*accurate determinations of the cell dimensions of crystals of ordinary and "heavy" ice (D_2_O)*’ through single-crystal X-ray diffraction (the study of the crystal structures of ice had been one of the main topics of Megaw’s PhD). A quite interesting aspect about this paper is the description of the set-up of the experiment, during which a drop of water was sealed into a glass capillary tube subsequently cooled into a mixture of acetone and carbon dioxide, until a good single crystal of ice was grown inside it. This set up was apparently (and intriguingly) the original inspiration for the now well-established procedure described below (see Section 7) of encapsulating biological crystals into a glass tube containing their mother liquor during X-ray diffraction measurements (Glazer, 2021 ▸). The unit-cell parameters of both ordinary and heavy ice thus obtained (see Fig. 6 ▸) showed very similar values within their standard uncertainties. This allowed a comparison of the ratio of the molecular volumes of crystalline D_2_O and H_2_O at 0°C (1.0014) with the ratio of the molecular volumes at 25°C (1.0034), and to infer that in the latter case, H_2_O shows a smaller volume per molecule than D_2_O, meaning that its molecules are more closely packed than those of deuterated water. [Since the publication of Megaw’s paper, it has been established that the O—H bond in H_2_O is longer than the O—D bond in D_2_O. See, for instance, Soper & Benmore (2008 ▸), which provides an investigation of the structures of heavy and light water at ambient conditions combining X-ray diffraction, neutron diffraction and computer simulations.]

The importance of these findings is summarized by the journal Editor in the section ‘Points from Foregoing Letters’, which indicates that ‘*Mr. (sic) H. D. Megaw reports that the crystal structure of "heavy" ice […] is the same as that of ordinary ice. […] His (sic) results further indicate that ordinary water (at room temperature) has its molecules more closely packed than "heavy" water, which has a more ice-like structure’.* Incidentally, the lack of influence of deuteration on the solid-state structure of water that emerges from Helen Megaw’s paper has also been analysed and observed in macromolecular crystallography (Fisher & Helliwell, 2008 ▸), where deuteration has been used, for instance, to determine the protonation state of ionizable amino acid side chains as a complement to X-ray crystallography (see *e.g.* Blakeley *et al.*, 2004 ▸).

*Brief biographical sketch:* Helen Dick Megaw (1907–2002) was an Irish crystallographer from Dublin. She first attended Queen’s University in Belfast before moving to Girton College, Cambridge, where she also received her doctoral degree. She worked as a teacher and as an industrial crystallographer at Philips Lamps Ltd before taking up a post at the Cavendish Laboratory in Cambridge. During her career, Megaw specialized in ferroelectricity in crystals, studied the structure of ice and solved the first structure of a perovskite. For these last two achievements, both an island (Megaw Island in Antarctica) and a perovskite-group mineral (Megawite) were named after her. She was also a consultant for the Festival Pattern Group of the Festival of Britain 1951, helping to establish a link between crystallography and design. In 1989 she was awarded the Roebling Medal from the Mineralogical Society of America. A short biography written by Mike Glazer (2018 ▸) can be found at: https://history.amercrystalassn.org/biography---megaw and https://ieeexplore.ieee.org/document/9336355.

## J. D. Bernal & D. Crowfoot (1934 ▸). *The initiation of protein X-ray crystallography*

This research article, entitled ‘*X-Ray Photographs of Crystalline Pepsin*’, concludes with this prophetic statement:

‘*Now that a crystalline protein has been made to give X-ray photographs, it is clear that we have the means of checking them and, by examining the structure of all crystalline proteins, arriving at far more detailed conclusions about protein structure than previous physical or chemical methods have been able to give*.’

Whilst crystals of proteins had been known for a very long time, since 1840 (see Giegé, 2013 ▸ for a historical overview), Bernal & Crowfoot (1934 ▸) showed for the first time X-ray diffraction from an ordered protein crystal. The fulcrum of their paper is that ‘*When examined in their mother liquor, they appear moderately birefringent and positively uniaxial, showing a good interference figure. On exposure to air, however, the birefringence rapidly diminishes. X-ray photographs taken of the crystals in the usual way showed nothing but a vague blackening*.’ The brilliant idea was to ‘*avoid alteration of the crystals, and this was effected by drawing them with their mother liquor and without exposure to air into thin capillary tubes of Lindemann glass. The first photograph taken in this way showed that we were dealing with an unaltered crystal. From oscillation photographs with copper Kα-radiation, the dimensions of the unit cell were found to be *a* = 67 Å, *c* = 154 Å, correct to about 5 per cent*.’ Furthermore, we read their comment about the pepsin crystal diffracting to high resolution, which they expressed in this way ‘*From the intensity of the more distant spots, it can be inferred that the arrangement of atoms inside the protein molecule is also of a perfectly definite kind*.’

The postal address of the authors at the end of their article is ‘Department of Mineralogy and Petrology, Cambridge’. The next year, Dorothy Crowfoot published her research article ‘X-Ray Single Crystal Photographs of Insulin’, a follow-up investigation to Bernal & Crowfoot (1934 ▸), and which is written under the postal address of the ‘*Department of Mineralogy, Oxford*’. In a curious twist, Dorothy Crowfoot remarks in her 1935 ▸ paper ‘*The (insulin) crystals prove to be perfectly stable in air (unlike pepsin) with unchanged birefring­ence and reflecting power, and it was accordingly possible to examine them dry by X-ray methods.*’ On a personal note, when JRH commenced his DPhil in Oxford in 1974 on determining the crystal structure of the enzyme 6-phospho­gluconate de­hydrogenase (Adams *et al.*, 1977 ▸), a crystal for X-ray diffraction data collection was mounted in the manner of Bernal & Crowfoot (1934 ▸) and not that of Crowfoot (1935 ▸). Indeed, the capillary method with a blob of the crystallization mother liquor on the protein crystal was the method in common use until flash freezing of a ribosome crystal became the required method (Hope *et al.*, 1989 ▸), and preferred also for other proteins for many years. For a historical overview, see Haas (2020 ▸). There is now somewhat of a resurgence of room-temperature protein crystallography for determining protein structure and dynamics in the context of the living organism (see *e.g.* Helliwell, 2020 ▸).

Both Bernal & Crowfoot (1934 ▸) and especially Crowfoot (1935 ▸) devote a sizeable proportion of their articles to discussing their unit-cell parameters, both the precision and whether integer multiples might be appropriate, and then the crystal packing. The actual crystal-structure determination of a protein crystal only became tractable with the introduction of the multiple isomorphous replacement method (Green *et al.*, 1954 ▸).

*Brief biographical sketches*:

John Desmond Bernal (1901–1971) was an Irish scientist who laid the foundation for a structural molecular biology using X-ray crystallography. He was also a polymath, publishing influential books on a wide range of subjects, notably *‘The Social Function of Science’* (Bernal, 1939 ▸), which JRH reviewed recently, including its historical importance (Helliwell, 2018 ▸). There is an extensive biography of Bernal’s life by Andrew Brown ‘*J. D. Bernal: The Sage of Science*’ (2005 ▸).

Dorothy Crowfoot (later married name, Hodgkin, adapted to become Dorothy Crowfoot Hodgkin), (1910–1994): Dorothy Hodgkin was awarded the Nobel Prize in Chemistry in 1964, the only British female scientist to be so honoured thus far. The Nobel Prize award citation was ‘for her determinations by X-ray techniques of the structures of important biochemical substances’. An extensive biography of Dorothy Hodgkin was written by Georgina Ferry (2014 ▸).

## E. W. Hughes (1941 ▸). *The first least-squares refinement of a crystal structure against its X-ray diffraction data*

In the history of crystal-structure analysis, a major methodological transition was the introduction by Hughes (1941 ▸) of least-squares model refinement against diffraction data.

**Figure 7:**
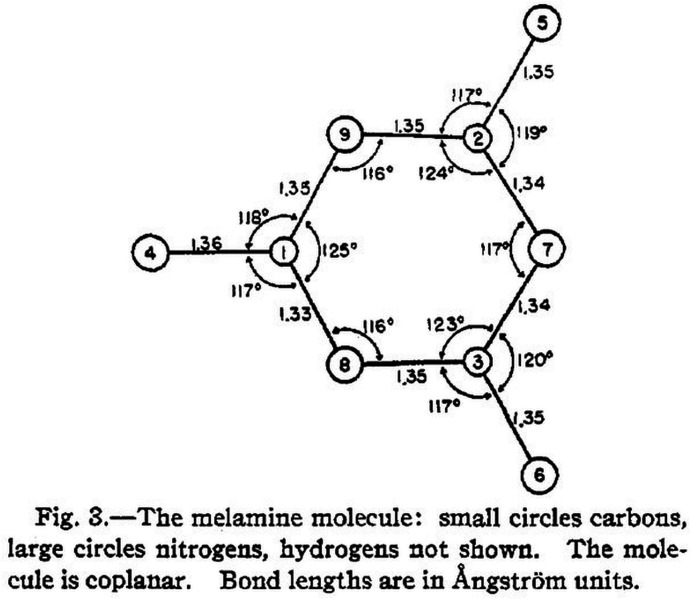
This figure of Hughes (1941 ▸) is shown with its original caption. On the precision of these bond distances shown in this figure Hughes (1941 ▸) remarks ‘*The displacements of the atoms from the average plane of the molecule are all within the limits of the probable error, which is estimated to be about ±0.015 Å for the position of an atom and about ±0.02 Å. for the length of a bond. It is thus not very likely that any bond length is in error by more than 0.05 Å.* Reprinted (adapted) with permission from Hughes, E. W. (1941 ▸). *J. Am. Chem. Soc.* **63**, 1737–1752. Copyright (1941) the American Chemical Society.

As Jim Ibers (2020 ▸) remarked in his ACA biographical sketch, ‘*Edward Hughes in the Pauling Group in 1941 was the first to apply the least-squares technique to the refinement of crystal structures*.’ Hughes (1941 ▸) used an ‘*International Business Machines Co. Tabulator using the Hollerith punched card system*’ instead of the manual Beevers–Lipson strips that he reported in his publication the year before (Hughes, 1940 ▸). It was in Hughes (1940 ▸) though that he mentioned the word ‘reliability’. This was with respect to the measured intensities rather than the molecular model. Whilst Hughes (1940 ▸, 1941 ▸) tabulates the measured structure-factor amplitudes and the corresponding values calculated from the molecular model, an overall residual was not calculated. Hughes (1941 ▸) emphasized the practical details of the calculation for melamine (Fig. 7 ▸), a crystal structure with nine non-hydrogen atoms, as follows: ‘*The cards were punched, verified, and the normal equations produced in slightly less than two days. The resulting normal equations consisting of eighteen simultaneous equations in the eighteen parameters were solved by an iteration method in about four hours.’* Interest in molecular model refinement was evidently stirred by the Hughes (1941 ▸) paper and other variants followed. Cruickshank (1952 ▸, 1960 ▸) compared the relationship between the Fourier and least-squares methods. The Fourier method was developed by Booth (1945 ▸, 1946 ▸, 1947 ▸). The method of Booth involved corrections to the atomic parameters in real space based on a difference-Fourier map. The procedure for crystal-structure analysis that is generally used today of course involves first of all a solution to the phase problem, then a difference-Fourier electron-density map to locate any missing atoms, or indicate disordered moieties, and finally a molecular model refinement as well as the addressing of *checkCIF* or PDB validation report alerts by the crystallographer (see, for example, the book by Giaco­vazzo *et al.*, 2002 ▸).

Hughes (1941 ▸) concludes as follows: ‘*Summary*. *The crystal structure of melamine has been investigated. The monoclinic unit has a = 10.54 Å, b = 7.45 Å, c = 7.25 Å, β = 112°2’. The space group is P2_1_/a, and there are four molecules per cell. A new method for refining parameters, based upon least squares, has been described and was used in conjunction with Fourier syntheses to locate the atoms.*’ He also has an acknowledge­ment: ‘*In conclusion I must thank Professor (Linus) Pauling*’.

*Brief biographical sketch:* E. W. Hughes (1924–1979) was based at Caltech in Pasadena, California, USA, when he published his 1940 ▸ and 1941 ▸ papers. He served as ACA President in 1954. An extensive description of the academic career and publications of E. W. Hughes can be found at https://pdf.oac.cdlib.org/pdf/caltech/hughese.pdf. There is an interview with Hughes at https://oralhistories.library.caltech.edu/296/1/Hughes%2C%20Edward_OHO.pdf. He was directly connected to Bragg, as well as Pauling. We quote from the latter, ‘*for a whole term, I had Bragg to myself, practically. We had a suite of offices together. And for a person just starting out in X-ray crystallography, to have Bragg at his elbow for five months was just unbelievable.’*

## J. M. Bijvoet, A. F. Peerdeman & A. J. van Bommel (1951 ▸). *Determination of the absolute configuration of optically active compounds by means of X-rays*

**Figure 8:**
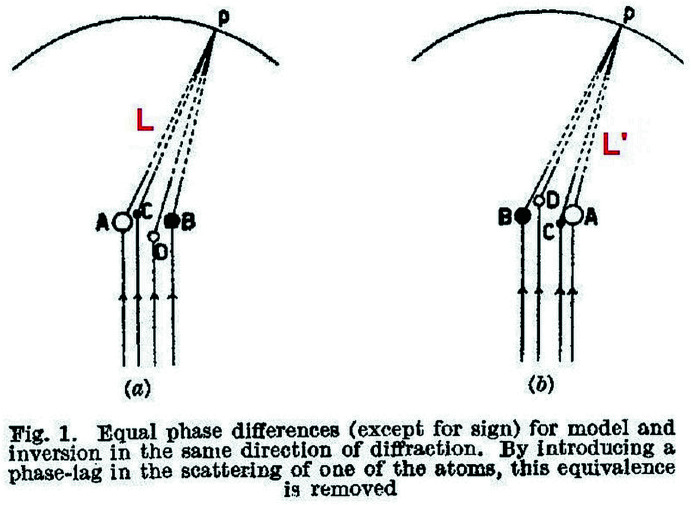
Path differences for two enantiomers of generic formula *ABCD*. Adapted from J. M. Bijvoet *et al.*. (1951 ▸). *Determination of the absolute configuration of optically active compounds by means of X-rays*. *Nature*, **168**, 271–272. Copyright Springer Nature. Reproduced with permission.

Determining the absolute configuration of enantiomers is a cornerstone of structural chemistry and, not surprisingly, a problem that was of very early interest for crystallographers. The paper by Bijvoet *et al.* ▸ precisely tackles this question. The heart of the problem is that ‘X-rays are not supposed to be able to determine absolute configuration as they measure the interatomic distances, which do not differ from model and inversion’ (Bijvoet *et al.*, 1951 ▸). Since the phase differences of the diffracted waves for two enantiomers are the same except for the sign (Fig. 8 ▸), the solution is the introduction of a ‘phase-lag’ to remove this equivalence.

If the wavelength of the incoming X-rays is *modified* to excite *just one atom* (for instance *A* in the example), the paths *L* and *L*′ connected with the diffracted waves from *A* in the two enantiomers are different, and the diffraction intensities will be also be different. Friedel’s law is completely broken and that finally allows the discrimination between a model and its inverse. It is impressive that these differences, very weak and difficult to measure, were detected at all. In this respect, in the foreword of the Proceedings of an IUCr conference on anomalous scattering held in 1974, Dorothy Hodgkin recollects that, ‘*I remember very well looking with Sir Lawrence Bragg at the 1951 ▸ paper from Professor Bijvoet’s laboratory which first gave experimental evidence of the ‘Bijvoet’ effect. Sir Lawrence said he found it difficult to believe such small differences in intensities could be measured reliably, but he had himself looked at the photographs in Professor Bijvoet’s laboratory in Utrecht and was convinced the effects were real’*. The interested reader can also refer to a related work (Bijvoet, 1954 ▸) reviewing the use of the anomalous scattering of X-rays combined with isomorphous replacement for the determin­ation of absolute configurations in crystals (a method that, incidentally, has played a fundamental role in the development of protein crystallography). In this paper, the determin­ation of the absolute configuration of a chiral compound through the measurement of anomalous dispersion at a single wavelength is treated in parallel with a more general problem, *i.e.* the determination of the phase of the diffracted ways in the Fourier method of X-ray analysis. Interestingly, this is also the focus of the paper by Okaya & Pepinsky ▸ (see Section 10), albeit in this case it is treated with the multiple-wavelength approach.

*Brief biographical sketch:* Johannes Martin Bijvoet (1892–1980) was a Dutch crystallographer who implemented the use of anomalous dispersion, combined with isomorphous replace­ment, to allow the structural determination of the absolute configuration of molecules. Bijvoet graduated in Chemistry and Physics from the Municipal University of Amsterdam in 1919, after the end of World War 1. He remained there for nearly ten years as assistant to his tutor, Professor A. Smits, who was also the ‘promotor’ for his doctoral degree on a thesis entitled ‘X-ray investigation of the crystal structure of lithium and lithium hydride’. From 1928 to 1939 he was a reader in crystallography and thermodynamics in Amsterdam, and successively moved to the van’t Hoff Laboratory of the State University of Utrecht where he remained until his retirement. He was President of the International Union of Crystallography from 1951 to 1954. Read more at https://royalsocietypublishing.org/doi/epdf/10.1098/rsbm.1983.0002 or Groenewege & Peerdeman (1983 ▸).

## Y. Okaya & R. Pepinsky (1956 ▸). *Solution of the phase problem by altering the X-ray wavelength*

The IUCr Pamphlet No. 8 entitled ‘*Anomalous dispersion of X-rays in crystallography. The contribution of resonance or dispersion effects to the atomic scattering factors’* by Caticha Ellis ▸ (1930–2003) provides a comprehensive account of the early decades of harnessing these effects prior to tuneable synchrotron radiation being available. See also Peerdeman (1975 ▸). The variation of X-ray wavelength was feasible in a very coarse manner by choice of metal for the anode in an X-ray tube. Thus, the stimulation of these anomalous dispersion effects of specific atoms was possible. It is feasible to use tube emission lines of different elements on a conventional source to do multiple X-ray wavelength phasing analysis. Hoppe & Jakubowski (1975 ▸) performed that experiment for the protein erythrocruorin using its iron atom. They used Ni and Co *K*α radiation to collect two data sets about the iron *K* edge (1.743 Å) and phases of the structure factors were determined with a figure of merit of 64% (mean phase error = 50°).

**Figure 9:**
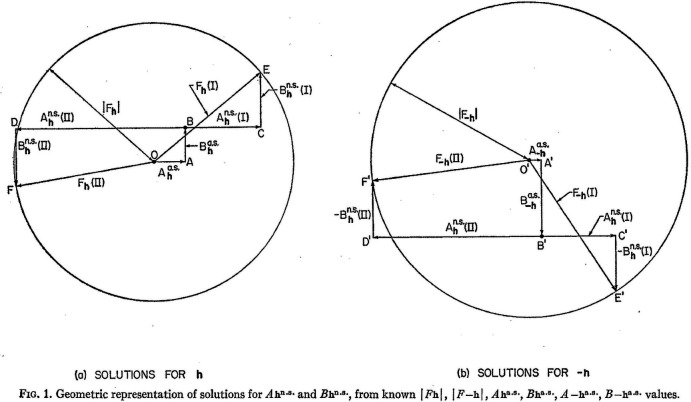
Figure with the original caption from the 1956 ▸ paper; n.s. refers to normal scattering and a.s. to anomalous scattering. We also need to quote from their main text: "*let us assume that the positions of the anomalous scatterers have been established by usual methods (e.g., from interpretation of Patterson maps, including if necessary joint Patterson maps obtained by altering the incident X-ray wavelength so that in one case no anomalous scattering occurs). Then Ah^a.s.^, Bh^a.s^, A-h^a.s^, and B-h^a.s^ are known; and of course |Fh|^2^ and |F-h|^2^ are known from the original intensity measurements….The phase problem for a non centric crystal, containing anomalous scatterers in known positions and normal scatterers in unknown positions, has now been reduced to the choice between [phase] solutions I and II.’* See also Helliwell (1984 ▸) Section 8.3 discussion and Figs. 51, 52 and 53 therein. Reprinted figure with permission from Okaya, Y. & Pepinsky, R. (1956 ▸). *Phys. Rev.* **103**, 1645–1647. Copyright (1956) by the American Physical Society.

As Caticha Ellis highlights, the attempts to define a theor­etical formalism for ‘solving structures directly by this phenomenon’ involved various researchers, with the first being Pepinsky and his collaborators (see references 14 to 27 of Caticha Ellis, 2001 ▸). Thus, Okaya & Pepinsky (1956 ▸) sought ‘to directly determine the phases of structure factors’. They assume that the positions of the anomalous scatterers have been determined by the Patterson (1934 ▸) method. At this stage, the phase determination of an individual structure factor is between two choices (I and II), which they illustrate with their Figure 1 (see Fig. 9 ▸). Their paper then offers several ideas to make a choice of phase between I and II for each reflection. Option 2 is to alter the X-ray wavelength so that ‘the anomalous scattering is avoided’, *i.e.* this moves the vectors in the left and right hand diagrams and has equivalent changes in the equations to solve the phase. However, the most helpful comment is in their remark to consider replacing the anomalous scatterer isomorphously with an atom of quite different normal scattering power. This suggestion is of course appealing in explaining the mathematics, but if used would defeat the object of the whole analysis to determine the phase of each structure factor without recourse to chemical treatment. What is missing from the paper, at this point, is an explicit treatment of the Δ*f*′ *versus* X-ray wavelength as well as the focus on the *f*′ effect, and its variation with X-ray wavelength.

Overall, this paper is an example of ‘science pull’ rather than ‘technology push’, which came eventually with the availability of tuneable synchrotrons (see chapter 9 of JRH’s book; Helliwell, 1992 ▸, paperback 2005). In the modern era, since the advent of synchrotron radiation, the field of protein crystallography has benefitted greatly from both the application of multiple X-ray wavelengths for phase determination and for the location and identification of metal atoms or ions. A major, quite general, initiative for phasing led by Wayne Hendrickson was the use of seleno­methio­nine in proteins, with the Se *K* edge conveniently placed at an X-ray wavelength of 0.98 Å to vary the selenium *f*′ and *f*′′ values with wavelength (Hendrickson *et al.*, 1990 ▸).

*Brief biographical sketch:* Ray Pepinsky (1912–1993). Quoting from the obituary in the IUCr Newsletter (Simon, 1994 ▸): ‘Ray Pepinsky obtained his doctorate in Crystallography under Zachariasen at the University of Chicago in 1940 and joined the Alabama Polytechnic Institute (API) as a faculty member in 1941. He took leave from the API to join a group of scientists at the MIT (Massachusetts Institute of Technology) Radiation Laboratory working on problems of national interest, including the development of radar during the Second World War. At Pennsylvania State University from 1949 to 1963 Ray carried out the studies in crystallography for which he is most famous’. (In this section we have expanded the quoted abbreviations of institutes or universities into their full titles.)

*Brief biographical sketch:* Y. Okaya. We were unable to find much information on Dr Okaya, even with the kind help of Dr Virginia Pett of the American Crystallographic Association. We did find out that he published crystal structures in the IUCr Journals whilst based at the IBM Watson Research Center, Yorktown Heights, New York, and also whilst at the Chemistry Department, State University of New York at Stony Brook, Stony Brook, New York. These were after his publications with Dr Pepinsky whilst at the Department of Physics, Pennsylvania State University.

## P. Coppens (1977 ▸). *Charge density analysis to elucidate the nature of chemical bonding*

Philip Coppens is acknowledged as one of the founders of charge-density analysis based on X-ray diffraction studies; he wrote an exhaustive textbook on this technique (Coppens, 1997 ▸) and several papers and reviews in later years to monitor its successive developments (see, for instance, Coppens, 1998 ▸, 2005 ▸, 2015 ▸; Koritsanszky & Coppens, 2001 ▸). For those approaching for the first time this broad and rich field of research, Coppens’ paper from 1977 ▸ presents very clearly several basic concepts, experimental considerations, and some practical examples on the type of information on chemical bonding that charge-density analysis can provide. The first part of the paper highlights the fundamental issue of how to determine experimentally the deformation effect produced on the electron density of free atoms upon bonding, which can be described by a function called *deformation density*. Since this function is the ‘*difference between the observed density and the density of all the free atoms in their electronic ground state centred at the atomic positions in the crystals*’, the precise and accurate evaluation of these densities from observed and calculated X-ray amplitudes is at the heart of the method and of its developments, prompting some fundamental considerations. (i) The density of the unbound atoms is a summation of calculated X-ray amplitudes of the free-atom functions, and during structural refinement atoms are assumed to have a spherical distribution *as if they were not bonded*. As a result, the least-squares procedure used to obtain the best fit between the calculated and the observed structure factors tends to reduce the deformation density to zero. This phenomenon had been already hinted at by W. H. Bragg (1920 ▸), who demonstrated that the properties of the carbon atom in diamond were based upon a tetrahedral and not a spherical form. In addition, while analysing the scattering of X-rays by microcrystalline graphite, Rosalind Franklin (1950 ▸) had noticed an anomalously high intensity for the (100) reflection. This was not compatible with a spherical distribution of the electron density, but could be explained by considering the three *sp* ^2^ electrons of the carbon atom ‘concentrated around the centre of the C—C bond rather than at the atomic nuclei’. Another very famous shortcoming deriving from the spherical distrib­ution is seen in bond lengths involving hydrogen atoms (Stewart *et al.*, 1965 ▸; Hamilton & Ibers, 1968 ▸; Churchill, 1973 ▸). The migration of electron density from the nuclear region of the H atom into the H—*X* bond introduces the well-known bias that makes the H—*X* bonds obtained *via* X-ray diffraction *ca* 0.12 Å shorter than those obtained by neutron diffraction (neutrons are scattered by atomic nuclei and not by the electrons). (ii) Neutron diffraction can thus provide an independent source of positional and thermal parameters, unbiased by the isolated-atom assumption, which can be determined separately from the bonding deformation on the electron valence density. (iii) To obtain the best quality electron densities with X-ray diffraction, it is important to collect as many intensities as possible, and at low temperature. The latter condition reduces the atomic vibrations in the crystals and thermal diffuse scattering, while increasing the order and the intensities of the reflections also at high θ angles.

The second part of the paper presents some case studies in which charge-density analysis provides information on the chemical bonding of different systems, by studying the so-called deformation density maps. These are a graphical representation of the electron-density differences in specific molecular planes and can help to detect and show the charge rearrangements taking place when bonding occurs. In Coppens’ words: ‘*A covalent chemical bond may be considered as resulting from the pairing of electrons of opposite spin, whose orbitals overlap in the bonding region. Theory indicates that the electron pairing leads to extra density in the bond compared to the density corresponding to the superposition of spherical atoms. This means that density should appear in the bonds in the deformation maps*’.

**Figure 10:**
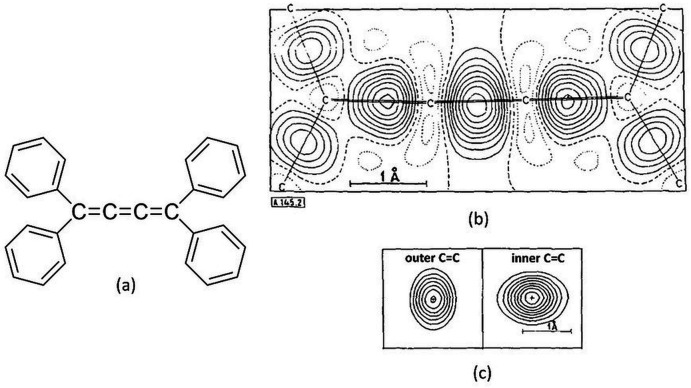
(*a*) Molecular sketch of tetra­phenyl­butatriene and (*b*) its deformation density map with contours at 0.10 e Å^−3^. Zero and negative contours are represented as broken and dotted lines respectively. (*c*) Deformation density sections perpendicular to the outer (left) and inner (right) C=C bonds through their centres. The charge in the outer C=C bond is elongated along the normal to the butatriene plane, while that in the inner C=C bond is elongated in the plane. Adapted from Coppens, P. (1977 ▸). *Experimental Electron Densities and Chemical Bonding.* *Angew. Chem. Int. Ed. Engl.* **16**, 32–40. Copyright Wiley-VCH GmbH. Reproduced with permission.

An example of such a map relative to tetra­phenyl­butatriene is given in Fig. 10 ▸, adapted from Figure 2 of the original paper. Density peaks between atoms are clearly visible, and the different elongations confirm that the densities of adjacent C=C bonds lie in mutually perpendicular planes, thus reflecting the predominance of *p*_*x*_ and *p*_*y*_ atomic orbitals in the *p* molecular orbital (Berkovitch-Yellin & Leiserowitz, 1975 ▸).

The paper concludes citing the possibility of obtaining quantitative results, such as net charges or dipole and quadrupole moments, from charge-density analyses. Interestingly, the example provided in this last section – the calculation of the numbers of electrons on an ion, atom or molecule, by integrating the electron-density function obtained from the X-ray data, introduces to the reader the important question of space partitioning. This term addresses the question of how to choose the volume for the integration and how to define the boundaries delimiting different ions, atoms or molecules in a crystal. This is also a fundamental issue in the analysis of theoretically calculated electron densities.

*Brief biographical sketch:* Philip Coppens (1930–2017) was a Dutch-born American crystallographer who developed the use of X-ray diffraction to perform charge-density analysis and also ‘photocrystallography’ (which we have not discussed), a technique that uses laser pulses timed to precede X-ray pulses to reveal the structure of highly reactive molecules in transient states, often referred to as ‘structural dynamics’. After receiving his PhD degree from the University of Amsterdam, he moved first to the Weizmann Institute and then to the Brookhaven National Laboratory. Finally, in 1968, he was appointed Professor in the Chemistry Department of the State University of New York at Buffalo, where he spent the rest of his career. In 2005 he was awarded the Ewald Prize by the International Union of Crystallography, and in 2011 he was named an American Crystallographic Association Fellow. A self-portrait describing his contributions to the field of crystallography can be found here: https://history.amercrystalassn.org/h_coppens_memoir.

## Conclusions

In writing short summaries of these articles, a challenging aspect was to convey to you, the reader, the context of the time when the research was undertaken, and when the articles were published. We hope that we have succeeded to a degree and have used this phrase ‘science pull and technology push’, where obvious, as a simple starting clarification of the context of research at the time. All of the examples show, to a greater or lesser degree, that the context of community developments and thinking are important to the individual researchers that we have highlighted. In more recent times, we can refer to the community-agreed validation reports of *checkCIF* and of the PDB as examples of community consensus. In our highlights there are examples of science advance by incremental change, albeit fairly large increments. There is at least one paradigm shift, most notably the first X-ray crystal structures determined by W. L. Bragg. In these matters then we can see that crystallography fits the different theories of the philosophy of science of how science advances: increments, paradigm shifts and consensus as well as team play and the insights of individuals.
